# Effect of Flaking and Precooking Procedures on Antioxidant Potential of Selected Ancient Cereal and Legume Flours

**DOI:** 10.3390/foods11111592

**Published:** 2022-05-28

**Authors:** Marco Consumi, Gabriella Tamasi, Claudia Bonechi, Marco Andreassi, Gemma Leone, Agnese Magnani, Claudio Rossi

**Affiliations:** 1 Department of Biotechnology, Chemistry and Pharmacy, University of Siena, Via A. Moro 2, 53100 Siena, Italy; marco.consumi@unisi.it (M.C.); claudia.bonechi@unisi.it (C.B.); marco.andreassi@unisi.it (M.A.); gemma.leone@unisi.it (G.L.); agnese.magnani@unisi.it (A.M.); claudio.rossi@unisi.it (C.R.); 2 National Interuniversity Consortium of Materials Science and Technology (INSTM), Via G. Giusti 9, 50121 Firenze, Italy; 3 Centre for Colloid and Surface Science (CSGI), Via della Lastruccia 3, 50019 Sesto Fiorentino (Firenze), Italy

**Keywords:** ancient cereals, legume flours, antioxidant properties, thermal pretreatment, extrusion pretreatment

## Abstract

Consumption of cereals (and particularly ancient cereals) is considered the base of a healthy diet, and all current dietary guidelines have cereals at the bottom of the nutrition pyramid. Together with cereals, legumes are an excellent source of nutrients and nutraceuticals. The effects of agroindustrial pretreatments (flaking and precooking processes) on the antioxidant potential of flours from ancient cereals and legumes were studied. The extraction of free hydrophilic phenolic compounds was carried out in a hydroalcoholic solvent mixture via an ultrasound-assisted process. Furthermore, the solid residue was successively hydrolyzed by an alkaline solution to extract the bound phenolic fraction. Both free and bound extracted fractions were then quantitatively characterized for total polyphenolic and flavonoid contents, and the antioxidant potential was determined by carrying out the ABTS and DPPH radical scavenging assays, expressing the results (in both cases) as the Trolox equivalent antioxidant capacity (TEAC/ABTS and TEAC/DPPH, respectively). The samples were also extracted in organic apolar solvents (acetone or water-saturated iso-butanol) to quantitatively characterize lipophilic antioxidant compounds and pigments. A discussion on the comparison of these analytical parameters of flours obtained from raw, flaked, and precooked cereals and legumes is reported revealing that (i) phenolic compounds are mainly present in the post-hydrolysis extract (bound fraction), (ii) the precooking process significantly reduced the concentration of antioxidants, (iii) the flaking process slightly increased the phenolic content, (iv) legumes were less influenced by pretreatments, suggesting the possibility of using legumes to enrich cereal foods.

## 1. Introduction

Recently, great efforts have been devoted to promote the use of composite flours, i.e., mixtures of flours from ancient cereals [1], and cereals belonging to different botanical origins to formulate fortified products. Several studies concerning the impact of mixing wheat with other cereals and legumes to enhance the nutritional properties of enriched products are ongoing [2,3]. As reported by the National Health and Medical Research Council, grains (in particular wheat, barley, maize, sorghum, rice, rye, millet, and oat) and foods made from them provide more than 56% of the energy and 50% of the proteins consumed by humans worldwide. Consumption of cereal grains is considered the base of a healthy diet, and all current dietary guidelines have cereals at the bottom of the nutrition pyramid as the largest component of the recommended daily intake [4]. Legumes and cereal grains are an excellent source of carbohydrates, dietary fibers, proteins, and minerals. In addition to these nutrients, both cereals and legumes are huge sources of phytoestrogens, antioxidants, and polyphenols. It is well-known that these minor components can act as protective agents against osteoarticular diseases [5], gastrointestinal cancers [6], cardiovascular disease [4,7] obesity, or diabetes thanks to their components that can act as direct free radical scavengers, cofactors of antioxidant enzymes, or as indirect antioxidants [8,9,10]. Legume flours are poorer in starch but richer in proteins and fibers than cereal flours. In particular, the starch content, which is about 60–80% in wheat, decreases to 40–60% in legumes. On the contrary, the protein content is around 20–30% in legumes, whereas it varies between 9 and 18% in wheat flours. Finally, the fiber amount is about 2% in wheat and reaches more than 10% in legumes [2].

Both legumes and cereals have to be subjected to processing before consumption, and these differences in composition make legumes and cereals differently susceptible towards pretreatments [11]. Processing allows removing toxic components, such as protease inhibitors, hemagglutinins, and growth inhibitors, making the final product more acceptable as food [12]. From the technological point of view, the term “flour” is usually used to identify ground material from wheat or other cereals (e.g., rye, maize, rice), seeds of non-cereals (e.g., buckwheat, sorghum, and millets), and legumes (e.g., soya beans and peas). Mature cereal grains are hard and rather indigestible as harvested from the plant unless they are steamed or soaked in water. Grinding or pounding the grains breaks the hard external coat and makes them more acceptable as foods. Flaking and heat pretreatment are two of the most commonly used processes because they can positively affect the nutritional profile of cereals and legumes and their technological properties related to subsequent uses. Thermal pretreatment is usually carried out via vapor steam treatment that allows reducing the numbers of viable microorganisms, even though it is not sufficient to sterilize the whole material, and inactivating the α-amylase enzymes that affect the degree of gelatinization. On the other hand, the flaking of cereals is usually carried out through compression of precooked grains or extruded pellets performed with rolls. This is a fundamental step in the production of a wide variety of ready-to-eat breakfast cereals, and also the first step of flour preparation (i.e., for baby food flours). Commonly, grains undergo steam vapor treatment in a closed steam chest at various pressures and times absorbing moisture; then, the product is pressed between pressure rollers to obtain thin flakes, and finally these can be ground. The improved digestive efficiency achieved by this process results from the fact that moisturization, heat, and flaking gelatinize (solubilize) some of the starches to render them more easily digestible. 

Indeed, even though high temperature can provoke decomposition of heat-labile compounds, mechanical treatment disrupting cell walls can increase the bioaccessibility and bioavailability of antioxidants and other nutraceutical compounds. The majority of antioxidants in grains are concentrated in the bran fraction and are covalently bound to indigestible polysaccharides, thus being not accessible to attack by enzymes in the human gastrointestinal tract, leading to low bioavailability [13].

In this context, the present study is aimed to: (i) achieve the optimal quality of final flours, (ii) characterize the antioxidant profile of raw cereal and legume flours and of flours from cereals and legumes that underwent different thermal treatments (precooking and flaking); and (iii) highlight the possible relation between flaking and precooking processes and their effects on the antioxidant potential of final products. It is known that nutrient losses occur in the preparation and cooking phases, and we believe that understanding how and why these losses occur can help consumers, chefs, and food processors limit losses and enhance the nutritional quality of food.

Although today the analytical protocols move forward from the basic TCP, DPPH, and TEAC assays to the forefront HPLC/MS-based analysis or in vitro antioxidant activity assay, these approaches require the availability of a well-equipped analytical laboratory often not present in small companies making niche products. Therefore, the potential data coming from more common and lower-technology instruments, such as UV–VIS spectrophotometry, though less specific, can be of great help to producers in the choice of the production process and, which is not less important, can be done internally in a very basic laboratory.

## 2. Materials and Methods

### 2.1. Chemicals

All the reagents were of analytical grade, purchased from Sigma-Aldrich (Milan): 2,2′-azino-bis(3-ethylbenzothiazoline-6-sulphonic acid) (ABTS), 2,2-diphenyl-1-picrylhydrazyl (DPPH), 6-hydroxy-2,5,7,8-tetramethylchroman-2-carboxylic acid (Trolox), aluminum trichloride (AlCl_3_), oxalic acid (C_2_H_2_O_4_), potassium persulfate (K_2_S_2_O_8_), sodium carbonate (NaCO_3_), sodium nitrite (NaNO_2_), sodium hydroxide (NaOH), hydrochloric acid (HCl), ferulic acid (C_10_H_10_O_4_), lutein (C_40_H_56_O_2_), α-tocopherol (C_29_H_50_O_2_), Folin–Ciocalteu reagent. All the solvents were of gradient grade, 99.9% HPLC purity: 1-butanol (*n*-BuOH), acetone (C_3_H_6_O), acetonitrile (MeCN), ethanol (EtOH), ethyl acetate (EtOAc), methanol (MeOH). Ultrapure deionized water was produced using an Acquinity P/7 purifier system (MembraPure GmbH, Berlin, Germany).

### 2.2. Plant Materials

All the flour samples from select ancient cereals and legumes (2017 crop harvest) were provided by an organic Tuscan farm. All the analyzed raw flours from cereals (mixture of soft wheats, einkorn, emmer, barley, oat, and millet), as well as from legumes (peas and chickpeas) were stone-milled starting from clean and slightly dehulled grains. Flour samples were also obtained after two different manufacturing processes: precooking and flaking of cereals and legumes (Table 1). The precooking process was under vapor steam. The flaking process was carried out starting from raw products that were hydrated in a stainless-steel vessel and underwent pressurized steam cooking, which was followed by air-drying to obtain the appropriate moisture for flaking. Then, steel rolls were used to flake the samples, which were finally milled.

### 2.3. Analytical Methods

#### 2.3.1. Starch and Crude Proteins Determination

The contents of starch and crude proteins were quantified using AACC International-approved methods 76-13 and 46-12, respectively [14].

#### 2.3.2. Extraction of Antioxidant Compounds 

*Extraction of hydrophilic phenolic compounds.* The extraction/hydrolysis processes were based on the previously published procedure [15] and optimized on the basis of specific matrices. Extraction of the free hydrophilic phenolic compounds was carried out starting from 0.2 g of flour in a test tube extracted twice with 5 mL of 80% cold aqueous ethanol (*v*/*v*). The suspension was homogenized by vortexing, and the extraction was ultrasound-assisted (10 min, 22 ± 2 °C; power, 120 W; sound frequency, 35 kHz; ultrasonic bath Sonorex Bandelin). The mixture was then centrifuged (5 min, 1882 g; centrifuge Thermo Electron Corporation PK 110), and all the supernatants were combined and dried under a gentle stream on nitrogen before freeze-drying (VirTis BenchTop Pro lyophilizer; −51 ± 2 °C, 1.5 ± 0.3 mbar, 48 h).

The solid residue was then treated to extract the bound hydrophilic phenolic compounds. It was suspended in 10 mL of 2N NaOH, homogenized by vortexing, and hydrolyzed in an ultrasonic bath (90 min, 40 ± 2 °C). After the alkaline hydrolysis, the resulting solution was acidified with 6N HCl up to pH 2. The aqueous solution was then extracted by ethyl acetate (three times, 5 mL each), shaken for 60 s, and centrifuged (5 min, 1882 g). The organic phase was finally evaporated to dryness under a gentle stream of nitrogen before storage at −20 ± 1 °C.

The dried residues obtained from the free and bound hydrophilic phenolic extractions were resolubilized in 1 mL 80% ethanol (*v*/*v*; 5 min, ultrasound-assisted) before subsequent analyses.

*Extraction of antioxidant lipophilic compounds.* Lipophilic antioxidants were extracted on the basis of the method previously reported [16], with slight modifications. An aliquot of 0.5 g of raw, flaked, or precooked flour (analytically weighed) was soaked with 0.5 mL of water for 5 min and extracted once with 2 mL and twice with 1.5 mL of pure acetone and vortexed (30 s) during each cycle; an ultrasound-assisted technique was used (5 min each cycle, 22 ± 2 °C). The supernatants were combined and the solvent was evaporated under a gentle stream of nitrogen overnight. The residue was then re-suspended in absolute ethanol and then stored at −20 ± 1 °C, before subsequent analyses. 

*Carotenoid extraction from cereal flours.* As already optimized and reported [17], 0.2 g of flour (analytically weighed) were extracted using 2 mL of water-saturated iso-butanol in an ultrasonic bath (15 min at 25 ± 2 °C) working in the dim light condition. The mixture was centrifuged (5 min, 1882 g), and the supernatants were collected and stored at −20 ± 1 °C before the analyses. 

*Chlorophyll extraction in pea flours.* An aliquot of 0.2 g of flour (analytically weighed) was extracted with 1 mL 80% acetone (*v*/*v*) by ultrasound-assisted extraction (5 min at 25 ± 2 °C). The mixture was centrifuged (5 min, 1882 g), and the supernatant was collected. The process was carried out three times until the resulting solvent became completely colorless, and the supernatants were combined (total volume of 3 mL).

#### 2.3.3. Antioxidant Compounds and Pigments Characterization

*Determination of the total polyphenolic content (TPC) in hydrophilic fractions.* The phenolic contents in the free and bound hydrophilic phenolic extracts of flours were determined using a colorimetric method as previously reported [18], with some modifications [19,20]. The sample solution (50 μL) was oxidized with Folin–Ciocalteu reagent (1.0 mL) and then neutralized with 20% (*w*/*v*) sodium carbonate solution (300 μL). The volume was adjusted to 2 mL with distilled water, thoroughly mixed, and incubated for 90 min in the dark. The solution was centrifuged (5 min, 1882 g), and the absorbance of the clear supernatants was measured at 765 nm. The calibration was set up using gallic acid standard aqueous solutions in the range of 0.5–10.0 mg/L, and the total phenolic content was calculated interpolating the calibration curve (R^2^ > 0.998) and expressing the results as milligrams of gallic acid equivalent per 100 g of the sample (mg GAE/100 g).

*Determination of the totalflavonoid content (TFC) in hydrophilic fractions.* Flavonoid contents in the free and bound hydrophilic phenolic extracts of flours (resolubilized in 1 mL of 80% ethanol, *v*/*v*) were determined by using the aluminum chloride colorimetric method as previously reported [21], with slight modifications. The sample solution (200 μL) was diluted with 800 μL of distilled water, and 60 μL of 10% sodium nitrite solution were added. The mixture was allowed to stand for 5 min before adding 120 μL of aluminum chloride (10%, *w*/*v*). After 5 min incubation, 400 μL of 1 M sodium hydroxide were added, and finally, the volume was adjusted to 2.0 mL, mixing thoroughly. The solution was incubated for 30 min in the dark at 22 ± 2 °C and the absorbance was measured at 510 nm. The calibration was set up using catechin standard aqueous solutions in the range of 2.5–40.0 mg/L, and the total flavonoid content was calculated interpolating the calibration curve (R^2^ > 0.998) and expressing the results as milligrams of catechin equivalent per 100 g of sample (mg CE/100 g).

*Antioxidant activity of hydrophilic fractions (TEAC/ABTS and TEAC/DPPH assays).* The Trolox equivalent antioxidant capacity (TEAC) for the free and bound hydrophilic phenolic extracts of flours was determined by using both the ABTS assay (TEAC/ABTS) and the DPPH assay (TEAC/DPPH), according to the protocols previously reported [22,23], with some modifications [19,20]. Briefly, the ABTS˙^+^ radical cation stock solution was generated, letting 140 mM K_2_S_2_O_8_ to react with 7 mM of ABTS in deionized water for 16 h in the dark. The stock solution was then diluted with ethanol to obtain the initial absorbance by ca. 0.750 at 734 nm. An aliquot of 20 μL of the properly diluted extract was added to 1 mL of the radical working solution and the final volume was adjusted to 1.1 mL. The system was carefully mixed and incubated for 30 min in the dark at 22 ± 2 °C. The quenching of the ABTS˙^+^ was determined as the absorbance decrease percentage at 734 nm (%*A*_734_), comparing the absorbances of the treated radical solution (*A_Sample_*) with respect to the blank (*A_Blank_*):

(1) $$\%Abs_{734}=\left(1-\frac{Abs_{Sample}}{Abs_{Blank}}\right)\times 100$$

The DPPH radical scavenging activity was evaluated starting from a mother solution 0.1 mM in MeOH, showing an initial absorption by ca. 0.700 at 517 nm. The extract (30 μL) was added by methanolic DPPH solution (1.0 mL) and the final volume was adjusted to 1.1 mL. The mixture was shaken vigorously and incubated for 30 min at 22 ± 2 °C, in dim-light conditions. The sample absorbance was then spectrophotometrically recorded at 517 nm. The quenching of the DPPH˙ was estimated by using a formula similar to that just above reported for ABTS assay (%*A*_517_).

In both cases the calibration curves were set up using Trolox standard aqueous solutions, in the range 5.0–20.0 μM, and the TEAC/ABTS and TEAC/DPPH values were calculated interpolating the calibration curve (R^2^ > 0.998), expressing the results as micromoles of Trolox equivalent per 100 g of sample (μmol TE/100 g).

*Antioxidant activity of lipophilic extract (α-TEAC/ABTS and α-TEAC/DPPH assays).* Measurements of the α-tocopherol equivalent antioxidant activity (α-TEAC) were assessed through both ABTS and DPPH assays, as just above reported. An aliquot of 30 μL of sample, standard solution, or ethanol as blank, was combined with 1.0 mL of ABTS˙^+^ or DPPH˙ and the final volume brought to 1.1 mL. After mixing thoroughly, the absorbance (at 734 and at 517 nm, for ABTS and DPPH assay, respectively) was recorded after 30 min of incubation in the dark at 22 ± 2 °C. In both cases, the antioxidant capacity of all extracts was compared to that of standard α-tocopherol solutions, used to set up the calibration (5.0–25.0 μM), and the α-TEAC/ABTS and α-TEAC/DPPH values were calculated interpolating the calibration curve (R^2^ > 0.998), expressing the results as micromoles of α-tocopherol equivalents per 100 g of sample (μmol αTE/100 g).

*Carotenoids determination in cereal flours.* Carotenoids were quantified as total yellow pigments, based on spectrophotometric determination, measuring the absorbance of the sample at 435.8 nm, and then expressed as milligrams of β-carotene equivalent per 100 g of flour sample (mg βCE/100 g; optical density at 435.8 nm of 1.0 mg in 100 mL water-saturated iso-butanol in a cuvette by 10 mm of optical path, being of 1.6632). The pigment content was also expressed as lutein equivalent per gram of flour, on the basis of the AACC reference method [14]. It was also calculated using a standard calibration curve of lutein solution in the range of concentration 0.25–8.00 mg/L from the absorbance readings at 450 nm. The results were then expressed as milligrams of lutein equivalent per 100 g of flour sample (mg LE/100 g).

*Chlorophylldetermination in peas flours.* Considering that the flaked and precooked peas underwent thermal treatment, possibly causing the degradation of the chlorophylls into pheophytins, the chlorophyll concentration in peas flour samples was determined by the procedure previously described [24], with some slight modifications. Two aliquots of each extract (1.940 mL) were analyzed: to the first, 60 μL of 80% acetone 80% (*v*/*v*) were added, and the second was treated with 60 μL of saturated oxalic acid in 80% acetone 80% (*v*/*v*) to allow the chlorophyll to pheophytin conversion (final volume, 2 mL). The solutions were stored in the dim light condition (22 ± 2 °C) for 3 h. Subsequently, the absorbances of both the acidified and non-acidified solutions were recorded at 662 and 645 nm, while measurements at 666 and 655 nm were conducted only for the acidified solutions. The chlorophyll a (Chl *a*), chlorophyll *b* (Chl *b*), and total chlorophyll (Chl *a* + *b*) concentrations were calculated using the conversion formula reported here:

(2) $$\left[Chl\;a\right]=25.38\Delta A_{662}+3.64\Delta A_{645}$$

(3) $$\left[Chl\;b\right]=30.38\Delta A_{645}-6.58\Delta A_{662}$$

(4) $$\left[Chl\;a+b\right]=18.80\Delta A_{662}+34.02\Delta A_{645}$$

where Δ_662_ and Δ_645_ represent the difference of the non-acidified (not converted) and acidified (converted) pigment solutions at 662 and 645 nm, respectively. These equations take into account the possible presence of pheophytin *a* and *b* in pea flour samples.

The total chlorophyll content (*Chl_t_*) of raw peas, considering any conversion of chlorophylls in pheophytins, was also estimated as follows:

(5) $$\left[Chl_{t}\;a\right]=20.65A_{666}-6.02A_{655}$$

(6) $$\left[Chl_{t}\;b\right]=32.74A_{655}-13.75A_{666}$$

(7) $$\left[Chl_{t}\;a+b\right]=6.90A_{666}+26.72A_{655}$$

The results were then expressed as milligrams per 100 g of flour sample (mg/100 g).

### 2.4. Statistical Analysis

Triplicate samples were collected and pretreated (extracted in triplicate), and finally triplicate analyses were performed for all the measurements, and the mean values and standard deviations were calculated and reported (mean ± SD; *n* = 27). The significant differences (*p* < 0.05) between the data were defined using one-way analysis of variance (ANOVA) followed by the appropriate multiple comparison Student’s test, Tukey’s, or Bonferroni test run on GraphPad Prism 8.0, used also for correlation tests. Regression studies were based on the Pearson correlation matrix, and the *p*-values were calculated at 95% confidence interval. All the graphical representations of the results were prepared using the Microsoft Office Excel 365 software.

## 3. Results and Discussion

### 3.1. Starch and Crude Proteins Determination

The contents of starch and crude proteins were quantified and the results are summarized in Table 2. 

From the statistical point of view, the values of both starch and crude proteins contents are not significantly affected by the precooking and/or flaking treatments. It is evident that flours from legumes revealed higher crude proteins contents when compared to the other samples. The values obtained in this study were comparable to those already reported.

### 3.2. Free and Bound Polyphenols and Flavonoids Contents

Polyphenols, and in particular flavonoids, represent the major antioxidant species in both cereals and legumes. The majority of studies quantifies only the free polyphenols fraction, considering it as total polyphenols [25], even if it has already been demonstrated that bound polyphenols strongly contribute to the total antioxidant activity [26]. Extracting bound polyphenols can be difficult because they are chemically linked to macromolecules, so they need to be hydrolytically cleaved, and this process can provoke alteration of the structure and function [27,28].

The polyphenolic and flavonoid contents, both in free and bound hydrophilic extracted fractions, are reported in Table 3. Extraction conditions were selected according to the methods previously optimized [25] (with some small modifications) choosing the adequate conditions to avoid, or at least reduce to the minimum, losses and degradation processes. The total phenolic (TPC) and flavonoid (TFC) contents were calculated as the sum of the two fractions.

Raw cereal flours showed a total polyphenolic content (as the sum of the free and bound fraction) in the range of 81 ± 2–144 ± 3 mg GAE/100 g, with the lowest and the highest values belonging to millet and the soft wheat mix, respectively. The total polyphenolic content in raw legume flours was about two or three times lower, ranging from 42 ± 1–56 ± 2 mg GAE/100 g, and these values are slightly lower than those previously reported (65 and 98 mg GAE/100 g in green peas and chickpeas, respectively [8]). The bound polyphenol fraction reaches a mean value of 71 ± 16 mg GAE/100 g for raw cereal flours (ranging between 52 ± 1 and 94 ± 2 mg GAE/100 g from millet to *Triticum dicoccum,* emmer), whereas a mean value of 12 ± 2 mg GAE/100 g was found for raw legume flours. For the free fraction, a smaller difference was revealed, averaging 50 ± 17 mg GAE/100 g for raw cereals and 37 ± 8 mg GAE/100 g for legumes.

Comparing the bound and the free fractions, different trends were revealed, resulting in a 1:1 ratio in most of the raw cereal flours, except for emmer and millet, in which the bound fraction represented 76% and 64% of the TPC, respectively. An opposite trend was observed for raw legume flours, where the bound fraction represented only 25% of the total phenolic content. These compounds are mostly present in the bran and the outer shell of grains so the low amount of bound phenolic acids quantified in legumes can be related to the fact that they are necessarily dehulled before being milled. The results for the free, bound, and total flavonoid contents (TFC) in untreated raw flour samples (Table 3) showed a general trend in agreement with that reported just above for the TPC. The TFC in raw cereal flours ranged between 33 ± 4 and 85 ± 2 mg CE/100 g, with the highest value belonging to raw barley and the lowest one—to raw millet, whereas the TFC in legume flours was about two times smaller, ranging between 22 ± 1 and 36 ± 1 mg CE/100 g. In barley and legume flours, the flavonoid contents in the free fractions were significantly higher than the respective bound fractions (averaging 65%; Tukey’s test, *p* < 0.05), in agreement with the dehulling process before being milled.

Flaking is a process that combines high temperature, high pressure, and high shearing conditions, and it can determine opposite effects on phenolic compounds in grains. It was demonstrated that it can cause decomposition of heat-labile polyphenolic compounds and polymerization of some polyphenolic compounds, thus decreasing the extractable polyphenolic content [29]. Nevertheless, breaking cell wall matrices and covalent polyphenol complexes, it can increase the accessibility of phenolic compounds, as clearly demonstrated by several studies [30,31]. The dominance of one effect or the other depends on several factors, mainly the extrusion processing parameters [32], but also the relative abundance of individual phenolic compounds can play a role due to their differences in chemical nature and stability under extrusion conditions [33]. A general statistically significant increase in total polyphenols after flaking pretreatment was revealed, in particular for the bound fraction (Bonferroni test, *p* < 0.05). An increase in the bound polyphenolic content of about 8.4% after extrusion in oat samples was revealed, in good agreement with a previous study that reported a 9% increase [34]. The highest increases were found for the bound fractions of the soft wheat mix, barley, and einkorn, i.e., 66%, 57%, and 46%, respectively. These high increases can be reasonable attributed to ferulic acid, which is the major component in wheat and that was predominant in the extrusion process as previously demonstrated [35]. Legumes showed the highest increase, of about four times, for both flaked peas and chickpeas with respect to raw flours The flaking process produced an increase of 37% in the total flavonoid content (TFC) of the oat flour and a slight but significant increase in the soft wheat mix, barley, and millet flours (about 6%; Bonferroni test, *p* < 0.05), whereas the einkorn and emmer flours significantly decreased by about 5% and 19%. The bran of grains is susceptible to the mechanical stress produced during the rolling process, that can contribute to the mechanical breakdown of cell walls, resulting in a better extractability of bound phenolic acids [28]. The effects were less pronounced for free phenolic acids, resulting in an increase or a decrease, depending on the different flour samples. This trend can be due to the combined effects of thermal treatment (thermal degradation) and mechanical treatment (better extractability) that occur during this process.

On the contrary, the precooking process produced a significant loss, both in the free and the bound fractions, in most of the samples, thus portraying the negative effect of thermal processing. Indeed, the TPCs in the precooked einkorn, barley, and chickpea flours were significantly lower with respect to the TPC in the flours obtained from untreated raw cereals (Bonferroni test, *p* < 0.05), resulting in a decrease of 72%, 39%, and 11%, respectively, probably due to the thermal degradation of polyphenols as already observed [36]. Among the cereals, only precooked millet showed a slight but significant increase in the polyphenol content in the bound fraction (21%, Bonferroni test, *p* < 0.05). A previous paper reported that the majority of phenolic compounds in five species of millet grains (Kodo, Foxtail, Proso, Little, Pearl) were quite stable after thermal treatment [37]. A significant increase was also observed in the precooked pea flours (28%) due particularly to the doubling-up of the TPC in the bound extracted fraction, while the free polyphenols did not reveal any significant variation (Bonferroni test, *p* > 0.05).

Finally, the millet flour surprisingly showed an increase of about four times of the TFC in the free extracted fraction. The remarkable increases revealed in this latter case, as well as in oat, can be reasonably explained by the effect of the flaking and precooking treatments on the free flavonoid fraction. This can be explained by the fact that, in case of short-term thermal treatments at a relatively low temperature, the vegetable matrix might retain its phenolic and flavonoid composition. Moreover, thermal processing may enhance the bioavailability, and therefore the extractability, of polyphenols by the breakdown of cellular structures and the inactivation of the oxidative and hydrolytic enzymes involved in their degradation [38,39]. As previously reported for the TPC, the increase accounted in the TFC of the flaked soft wheat mix and barley flours could be due to the mechanical stress produced during the rolling process. On the contrary, legume flavonoid fractions showed a low sensitivity to precooking/thermal‘ treatment.

### 3.3. Antioxidant Capacity by the ABTS and DPPH Assays

The Trolox equivalent antioxidant capacity (TEAC) was assessed by the ABTS (TEAC/ABTS) and the DPPH (TEAC/DPPH) methods, in the free and bound antioxidant extracts. The total antioxidant capacity was calculated as a sum of the free and the bound fractions. The results are summarized in Table 4. 

Polyphenols are considered as the main class of compounds responsible for the antioxidant properties in cereals and legumes. The total antioxidant capacity, indeed, followed the same trend discussed for the TPC and TFC, revealing total values (as the sum of the free and the bound fractions) in raw flours in the ranges of 262 ± 7–1091 ± 7 and 137 ± 2–374 ± 6 µmol TE/100 g for the ABTS and DPPH assays, respectively. In general, the total TEAC determined by the ABTS assay showed higher values than the DPPH assay.

This can be explained by the higher stability (and thus lower reactivity) of the DPPH radical with respect to the ABTS radical [40]. The different reactivity of the ABTS and DPPH radicals appeared more evident analyzing the TEAC in free and bound antioxidant fractions. While the results obtained by the DPPH assay confirmed results previously discussed for the TPC and TFC, the ABTS assay highlighted a much higher antioxidant capacity in the extracts obtained after alkaline hydrolysis. Except for legume flours, in which the bound antioxidant fraction corresponded to about 30% of the total TEAC, in cereal samples, it ranged from 64% in raw barley to 94% in raw emmer. Moreover, comparing the results obtained with the two different methods is interesting to notice that the free ABTS TEAC was only two times higher on average with respect to the free DPPH TEAC, while the bound ABTS TEAC was up to four times higher than the bound DPPH TEAC.

These findings can be explained considering the different mechanisms of reaction of the ABTS and DPPH radicals. Furthermore, the quenching capacity of polyphenols is strictly related to their chemical structure. Ferulic acid is one of the main phenolic acids in whole grains. Its content may differ between cereals, but in any case, it is mainly present in the bound insoluble form (>93%) [26]. Ferulic acid is also linked via an ester bond to arabinoxylans, which are among the main components of cereal dietary fibers. The dimerization of ferulates, with formation of diferulates, is involved in the cross-linking of these carbohydrates, providing chemical and physical protection of the cereal kernel [41]. Hence, one of the main effects of alkaline hydrolysis on cereal flours is to allow the extraction of the insoluble ferulic acid fraction by hydroalcoholic solutions. A previous study reported a higher reactivity of ferulic acid with ABTS˙^+^ than with DPPH˙ [42]. This different quenching capacity can justify the high ABTS TEAC values obtained in the bound fraction, while the slight difference detected in the nonhydrolyzed extracts by the two different methods underlined the fact that ferulic acid is a minor component of the free phenolic fraction.

For the same reasons as just mentioned above, the ABTS TEAC of legume flours resulted in an about one order of magnitude lower bound antioxidant fraction if compared with the values of cereal flours, while the TEAC of the free antioxidant extracts showed only slight differences with both the methods.

Due to the great variability in the antioxidant profile of vegetal matrices, it is strongly recommended to use at least two different methods to assess the antioxidant capacity [43]. In general, none of the two methods used in this study can be considered more suitable for the determination of the antioxidant capacity, but the ABTS and DPPH radicals with different reactivity give complementary information useful in the interpretation of results. 

**Figure 1:**
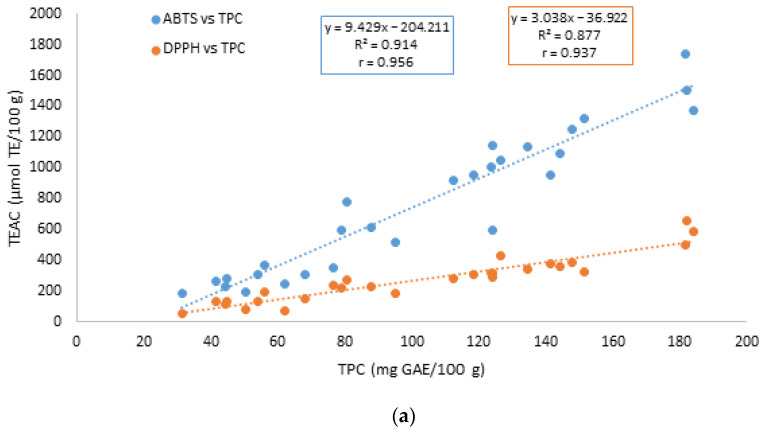

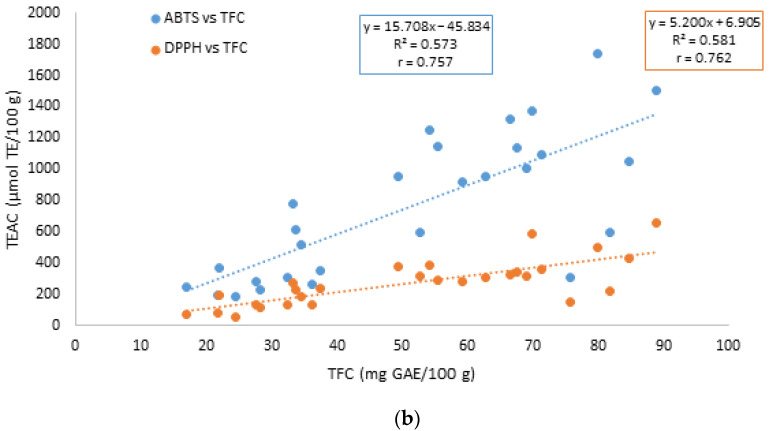
Linear regression of the total antioxidant capacity (TEAC) determined using the ABTS and DPPH assays versus (**a**) the total phenolic content (TPC) and (**b**) the total flavonoid content (TFC).

The discussion of the results for the raw cereal and legume flours can also be extended to the pretreated ones. The total antioxidant capacity detected by the ABTS and the DPPH methods was correlated with the TPC and TFC for all the samples, and the linear regressions are reported in Figure 1.

As reported, higher TEAC values corresponded to higher TPC and TFC, and the samples showing a lower TEAC exhibited relatively lower TPC and TFC. However, the correlation between the antioxidant activity revealed by the two assays (ABTS and DPPH) and TPC (r = 0.956 and 0.937, respectively) was stronger with respect to the correlation with the TFC (r = 0.757 and 0.762, respectively), suggesting a lower contribution of flavonoids to the total antioxidant activity. The TPC was slightly better correlated with the ABTS TEAC, the TFC—with the DPPH TEAC. The Pearson correlation coefficients (r) of the parameters measured in the hydrophilic free, bound, and total antioxidant fractions (phenolic content, flavonoid content, and ABTS TEAC and DPPH TEAC) are summarized in Table 5.

In the free antioxidant fraction, the flavonoid content was weakly correlated with the phenolic content (r = 0.378), indicating a low concentration of these compounds, also confirmed by the weak correlation between the flavonoid content and the DPPH TEAC (r = 0.477) and the nonsignificant correlation with the ABTS TEAC (*p* > 0.05). In the bound fraction, flavonoids showed a very strong correlation with polyphenols, ABTS TEAC, and DPPH TEAC (r = 0.884, 0.902, and 0.898, respectively), even though there were other compounds contributing to the bound antioxidant capacity, as indicated by the higher correlation of the phenolic content with both the ABTS TEAC and DPPH TEAC (r = 0.961 and 0.951, respectively). In the free, bound, and total fractions, ABTS TEAC and DPPH TEAC were significantly highly correlated, demonstrating that the ABTS and DPPH assays are suitable to be used together to measure the antioxidant capacity. It is important to recall that polyphenols and flavonoids are not the only classes of compounds able to scavenge the ABTS and DPPH radicals, but several other antioxidants can be found in vegetable matrices. Moreover, it must be taken into account that antioxidants influence each other synergistically or antagonistically when they are present in a complex mixture.

### 3.4. Carotenoid Content and Antioxidant Activity Quantification by α-TEAC in Lipophilic Extracts

Carotenoids are among the most abundant types of naturally occurring fat-soluble pigments. High intake of carotenoids has been associated with a lowered incidence of eye diseases, such as age-related macular degeneration (AMD), cataracts, and retinitis pigmentosa [44,45]. These yellow pigments content, expressed as mg of lutein and β-carotene equivalent per 100 g of flour sample, was quantified and reported in Table 6. 

As expected, the most colored ones have higher levels of both lutein and β-carotene. Unlike the data previously reported [46] that highlighted a higher amount of lutein in comparison with β-carotene, in the samples analyzed in the present work, the β-carotene content was significantly higher (Bonferroni test, *p* < 0.05) than that of lutein for both cereals and legumes. However, lutein is the predominant carotenoid in wheat accounting for 80–90% of the total carotenoids, while other carotenoids, such as β-carotene, zeaxanthin, and lutein esters, are present only in small amounts. β-Carotene was found to represent only 1% of the total carotenoids in wheat species [46]. For that reason, the total carotenoid content (TCC) was determined following AACC International-approved method 14-60.01, expressing the results as mg of lutein equivalent per 100 g of sample (mg LE/100 g; Table 6). Raw einkorn flours showed the highest amount of carotenoids (0.837 ± 0.006 mg LE/100 g), whereas the other raw cereal flours revealed lower values, in the range of 0.238 ± 0.003–0.562 ± 0.005 mg LE/100 g. In legume flours, the TCC showed the highest values, ranging between 0.934 ± 0.002 and 2.286 ± 0.002 mg LE/100 g. 

The flaking process brought about a significant decrease in the TCC (Bonferroni test, *p* < 0.05) of lutein ranging from 22% in emmer up to 90% in millet detected in agreement with a decrease in lipophilic antioxidants ranging between 63% and 95% in extruded oat, barley, wheat, rye, and buckwheat as revealed in other studies [35,47]. A superimposable trend was recorded for β-carotene. The soft wheat mix represents an exception with a revealed increase of 32%.

The fate of lipid-soluble antioxidants during thermal treatments was previously investigated and reported with different results and conclusions [48,49]. Thermal treatments can affect the carotenoid content in different ways depending on the time and thermal conditions used. In general, thermal processing may decrease the carotenoid content by degradation and isomerization, but at the same time can facilitate the extractability and the bioavailability of these compounds through the disruption of the food matrix, cell walls and membranes. Moreover, the behavior of each carotenoid may vary depending on its chemical structure and the food matrix in which it is contained [50]. As previously highlighted [48], steaming of raw grains can bring about both an increase or a decrease in carotenoids depending on the prevalence of one or the other of the two following effects. The loss of the lipid structure and the changes in the lipid distribution pattern together with the release of some bound molecules from cellular components can be observed with a consequent increase in carotenoid contents. In addition, steaming treatment can also bring about a strong migration of carotenoids from the bran into the flour. On the other hand, some heat-sensitive vitamin E isomers undergo degradation. The extent of each effect depends on the species, cultivar, and the specific thermal conditions [48]. According to these comments, it was found that the precooking process produced a significant increase in the TCC content (expressed as mg LE/100 g) of the chickpea, barley, and einkorn flours (11%, 21%, and 51%, respectively), while the precooked millet and pea flours showed a decrease of 61% and 36%, respectively. 

Measurements of the antioxidant capacity in the lipophilic extracts, expressed as α-tocopherol equivalents (α-TEAC), were carried out by both the ABTS and DPPH assays (Table 6), and the results obtained for all the samples were correlated with the TCC. Surprisingly, very weak nonsignificant correlations were found both with the ABTS α-TEAC and DPPH α-TEAC assay values (*p* > 0.05), suggesting that there is no relationship between the carotenoid content and the radical scavenging capacity of lipophilic extracts obtained by selecting acetone as the solvent because of its capacity to extract a broad range of lipophilic antioxidants. This can be explained considering the remarkably different antioxidant profiles of the analyzed samples.

As previously mentioned, lutein is the predominant carotenoid in wheat, while other carotenoids are present in lower amounts. Besides carotenoids, it is known from literature that *Triticum* species contain other powerful lipophilic antioxidants, such as vitamin E, which includes four tocopherol and tocotrienol homologs (α-, β-, γ-, δ-), alkylresorcinols, in which whole wheat flour is particularly rich since they exclusively occur in the intermediate outer layer of kernels, and steryl ferulates, which are esters of phytosterols with ferulic acid mainly concentrated in the bran of wheat kernels. Lutein and alkylresorcinols generally do not scavenge the DPPH radical, while all lipophilic antioxidants react in the ABTS assay [15].

Barley had the lowest TCC (0.284 ± 0.003 mg LE/100 g); however, it showed the highest α-TEAC with both the ABTS and DPPH assays (225 ± 3 and 99 ± 4 µmol αTE/100 g, respectively). It is reported that barley has a carotenoid profile different from that of wheat species, characterized by a high content of zeaxanthin, comparable to that of lutein, corresponding to 50% and 42% of the TCC, respectively [51]. On the contrary, lutein is reported to be the predominant carotenoid in legumes, accounting for 90% of the TCC in peas, while it corresponds to 54% in chickpeas, followed by the 41% of zeaxanthin [52]. With this in mind, it can be suggested that, besides carotenoids, other classes of compounds should be considered to describe the lipophilic antioxidant capacity of cereal and legume flours.

### 3.5. Chlorophylls Determination in Pea Flours

Several spectrophotometric quantitative methods are commonly used to determine the chlorophyll content in foodstuffs. One of the most commonly used allows the determination of chlorophylls by the direct measurement of absorbances at the maximal absorption of chlorophylls *a* and *b* [53]. However, this method is not directly applicable to systems in which pheophytins (products of chlorophyll degradation) may be present, for example, because of thermal food treatments. In this case, another method, proposed for the first time by Vernon [24], is more appropriate, which allows quantifying the chlorophylls actually present in the samples, taking into account the extent of conversion of chlorophylls to pheophytins. Using this method, it is also possible to estimate the chlorophylls originally present in the samples, before they were subjected to pretreatments, assuming that chlorophyll concentrations correspond to those of pheophytins quantified in the extracts acidified with oxalic acid.

In the present study, only pea flours showed a significant amount of chlorophylls. The results for the actual chlorophyll contents in pea flours (raw, flaked, and precooked) and the estimated chlorophyll content in peas (actual + degraded) before they were subjected to pretreatments (flaking and precooking) and the milling process are summarized in Table 7. 

It is easily understandable that the thermal treatment occurring during the flaking and precooking processes degraded the chlorophyll pigments, promoting the pheophytin conversion. Both pretreatments produced almost a complete degradation of Chl *a* (ca. 94%), from 3.70 ± 0.07 to 0.21 ± 0.03 mg/100 g in flaked peas and from 3.64 ± 0.01 to 0.236 ± 0.005 mg/100 g in precooked peas. Regarding Chl *b*, the effect of the flaking process was more pronounced than that of the precooking one, producing a decrease of 98% (from 1.71 ± 0.01 to 0.041 ± 0.001 mg/100 g) and 89% (from 1.86 ± 0.01 to 0.21 ± 0.01 mg/100 g), respectively. A slight significant decrease of about 30% was also detected in the raw pea flour for both Chl *a* and *b* (from 3.96 ± 0.07 to 2.9 ± 0.1 mg/100 g and from 2.60 ± 0.02 and 1.79 ± 0.01, respectively), probably due to the fact that the milling process also produces certain heating, thus promoting a partial degradation of chlorophylls.

## 4. Conclusions

Select samples of flours from ancient cereals and legumes were analyzed with the aim of profiling how their nutraceutical potential can be affected by agroindustrial pre-treatments, such as flaking and precooking. Hydrophilic (free and bound) and lipophilic fractions were properly extracted and the determination of the total polyphenolic and flavonoid contents, the Trolox equivalent antioxidant capacity (TEAC), as well as pigments contents were carried out via spectrophotometric assays. It has been revealed that phenolic compounds are mainly present in the bound insoluble form and that the precooking process appears to significantly lower the concentration of antioxidants. On the contrary, flours from flaked cereals and legumes showed, on average, slightly higher phenolic contents, even though differences in the antioxidant activity were usually less marked, probably because of mechanical compression forces. Furthermore, considering the nutritional (starch and crude proteins) and antioxidant (nutraceutical) profiles of legumes, these seemed to represent a possible matrix to enrich cereal foods, allowing fortification of the final product. Gluten-free legume flours, especially in intolerant people, may be considered as a viable alternative to common millet (and maize), with the advantage of providing the highest carotenoid content.

## Figures and Tables

**Table 1:** List of collected and analyzed flour samples obtained from select ancient cereals and legumes.

Flour from Raw Materials	Flour from Flaked Materials	Flour from Precooked Materials
**Cereals**		
Soft wheat mix ^1^	Flaked soft wheat mix ^1^	
Einkorn (*Triticum monococcum)*	Flaked einkorn	Precooked einkorn
Emmer (*Triticum dicoccum)*	Flaked emmer	
Barley	Flaked barley	
Oat	Flaked oat	
Millet	Flaked millet	Precooked millet
**Legumes**		
Peas	Flaked peas	Precooked peas
Chickpeas	Flaked chickpeas	Precooked chickpeas

^1^ This sample was a mixture of Tuscan ancient grain varieties: Sieve, Gentil Rosso, Verna, Frassineto, and Andriolo.

**Table 2:** Starch and crude proteins contents in the flours, reported as mean ± SD (*n* = 9) and expressed as g/100 g.

Flour/Processing	Starch (g/100 g)	Crude Proteins (g/100 g)
**Cereals**		
Soft wheat mix/raw	70 ± 14	10 ± 2
Soft wheat mix/flaked	68 ± 14	8 ± 2
Einkorn/raw	63 ± 13	12 ± 2
Einkorn/flaked	61 ± 12	9 ± 2
Einkorn/precooked	61 ± 12	12 ± 2
Emmer/raw	63 ± 13	12 ± 3
Emmer/flaked	66 ±13	12 ± 2
Barley/raw	69 ± 14	8 ± 2
Barley/flaked	66 ± 13	9 ± 2
Barley/precooked	70 ± 14	9 ± 2
Oat/raw	68 ± 14	8 ± 2
Oat/flaked	62 ± 12	13 ± 2
Millet/raw	68 ± 14	12 ± 3
Millet/flaked	72 ± 14	12 ± 2
Millet/precooked	68 ± 14	11 ± 3
**Legumes**		
Peas/faw	51 ± 10	21 ± 3
Peas/flaked	54 ± 11	19 ± 2
Peas/precooked	59 ± 12	16 ± 2
Chickpeas/raw	50 ± 9	23 ± 3
Chickpeas/flaked	45 ± 9	21 ± 3
Chickpeas/precooked	48 ± 10	21 ± 3

**Table 3:** Free and bound polyphenolic and flavonoid contents in the hydrophilic extracts, reported as mean ± SD (*n* = 27). Values in brackets represent the contribution of free and bound fractions to the total polyphenolic (TPC) and flavonoid (TPF) contents, that can be calculated as sum of the two fractions.

Flour/Processing	Total Polyphenolic Content (TPC, mg GAE/100 g)		Total Flavonoid Content (TFC, mg CE/100 g)	
	Free	Bound	Free	Bound
**Cereals**				
Soft wheat mix/raw	65.5 ± 1.1 (45%) ^aA^	78.7 ± 0.3 (55%) ^aA^	36.2 ± 2.4 (51%) ^aA^	35.2 ± 0.2 (49%) ^aA^
Soft wheat mix/flaked	51.3 ± 2.1 (28%) ^B^	130.5 ± 0.8 (72%) ^B^	30.0 ± 1.4 (38%) ^B^	50.0 ± 1.9 (62%) ^B^
Einkorn/raw	54.0 ± 1.3 (48%) ^bA^	58.4 ± 0.1 (52%) ^bA^	29.4 ± 0.4 (50%) ^bA^	29.9 ± 0.1 (50%) ^bA^
Einkorn/flaked	38.5 ± 3.2 (31%) ^B^	85.5 ± 0.6 (69%) ^B^	30.0 ± 0.7 (57%) ^A^	22.7 ± 1.7 (43%) ^B^
Einkorn/precooked	14.0 ± 1.2 (45%) ^C^	17.3 ± 0.8 (55%) ^C^	10.1 ± 0.2 (41%) ^B^	14.4 ± 1.7 (59%) ^C^
Emmer/raw	29.9 ± 1.0 (24%) ^cA^	93.7 ± 2.0 (76%) ^cA^	30.5 ± 3.9 (44%) ^bA^	38.5 ± 0.5 (56%) ^cA^
Emmer/flaked	42.9 ± 0.7 (35%) ^B^	81.2 ± 1.8 (65%) ^B^	24.0 ± 0.7 (43%) ^B^	31.5 ± 1.3 (57%) ^B^
Barley/raw	65.2 ± 3.7 (52%) ^aA^	61.2 ± 0.2 (48%) ^cA^	54.5 ± 1.0 (64%) ^cA^	30.2 ± 0.8 (36%) ^bA^
Barley/flaked	85.6 ± 2.5 (47%) ^B^	96.3 ± 3.2 (53%) ^B^	53.4 ± 1.0 (60%) ^A^	35.5 ± 1.7 (40%) ^B^
Barley/precooked	57.4 ± 2.4 (75%) ^C^	19.2 ± 0.4 (25%) ^C^	34.1 ± 1.7 (91%) ^B^	3.5 ± 0.1 (9%) ^C^
Oat/raw	58.5 ± 0.8 (41%) ^dA^	83.1 ± 0.3 (59%) ^eA^	17.3 ± 0.2 (35%) ^dA^	32.0 ± 1.0 (65%) ^dA^
Oat/flaked	61.3 ± 0.1 (41%) ^B^	90.1 ± 2.4 (59%) ^B^	32.2 ± 0.2 (48%) ^B^	34.4 ± 0.6 (52%) ^B^
Millet/raw	29.1 ± 0.9 (36%) ^cA^	51.5 ± 1.2 (64%) ^fA^	11.2 ± 4.1 (34%) ^eA^	22.0 ± 1.3 (66%) ^eA^
Millet/flaked	34.0 ± 3.6 (36%) ^A^	61.0 ± 0.4 (64%) ^B^	14.8 ± 0.7 (43%) ^B^	19.9 ± 1.5 (57%) ^B^
Millet/precooked	16.5 ± 0.3 (21%) ^B^	62.4 ± 1.1 (79%) ^B^	57.2 ± 2.2 (70%) ^C^	24.7 ± 0.5 (30%) ^C^
**Legumes**				
Peas/raw	30.9 ± 0.9 (74%) ^cA^	10.6 ± 0.2 (26%) ^gA^	21.3 ± 0.2 (59%)^fA^	14.9 ± 0.1 (41%) ^fA^
Peas/flaked	26.9 ± 3.9 (61%) ^A^	56.1 ± 2.9 (39%) ^B^	14.0 ± 0.6 (49%)^B^	24.6 ± 0.9 (51%) ^B^
Peas/precooked	32.2 ± 0.5 (60%) ^A^	21.8 ± 1.1 (40%) ^C^	20.2 ± 0.6 (62%)^A^	12.3 ± 0.1 (38%) ^C^
Chickpeas/raw	42.6 ± 1.0 (76%) ^eA^	13.5 ± 1.2 (24%) ^hA^	15.7 ± 1.3 (71%)^dA^	6.3 ± 0.5 (29%) ^gA^
Chickpeas/flaked	42.8 ± 4.2 (69%) ^A^	66.0 ± 1.4 (31%) ^B^	13.5 ± 0.1 (80%)^B^	20.2 ± 1.5 (20%) ^B^
Chickpeas/precooked	34.5 ± 0.2 (69%) ^B^	15.8 ± 0.3 (31%) ^C^	14.2 ± 0.8 (65%)^AB^	7.6 ± 0.1 (35%) ^C^

Within the same column, the values marked with the different letters are significantly different: lowercase letters compare the significant differences between raw flours (Tukey’s test; *p* < 0.05), and capital letters compare the significant differences between raw/flaked/precooked flours for the same species (Student’s *t*-test or Bonferroni test; *p* < 0.05).

**Table 4:** Free and bound Trolox equivalent antioxidant capacity (TEAC) determined by the ABTS and DPPH assays in the hydrophilic extracts, reported as mean ± SD (*n* = 27). The values in brackets represent the contribution of free and bound fractions to the total antioxidant capacity that can be calculated as the sum of the two fractions.

Flour/Processing	TEAC/ABTS (μmol TE/100 g)		TEAC/DPPH (μmol TE/100 g)	
	Free	Bound	Free	Bound
**Cereals**				
Soft wheat mix/raw	337.0 ± 7.1 (31%) ^aA^	754.3 ± 1.6 (69%) ^aA^	157.8 ± 1.3 (43%) ^aA^	205.3 ± 11.1 (57%) ^aA^
Soft wheat mix/flaked	418.2 ± 16.1 (24%) ^B^	1324.0 ± 24.6 (76%) ^B^	197.1 ± 9.7 (40%) ^B^	299.8 ± 0.4 (60%) ^B^
Einkorn/raw	271.8 ± 3.0 (29%) ^bA^	651.1 ± 25.3 (71%) ^bA^	113.3 ± 2.6 (40%) ^bA^	172.1 ± 2.5 (60%) ^bA^
Einkorn/flaked	160.4 ± 0.4 (27%) ^B^	439.0 ± 1.2 (73%) ^B^	154.0 ± 0.4 (48%) ^B^	165.2 ± 0.5 (52%) ^B^
Einkorn/precooked	118.3 ± 3.2 (65%) ^C^	64.2 ± 0.3 (35%) ^C^	38.6 ± 1.0 (75%) ^C^	12.8 ± 0.1 (25%) ^C^
Emmer/raw	55.9 ± 3.5 (6%) ^cA^	949.8 ± 33.0 (94%) ^cA^	51.5 ± 3.9 (16%) ^cA^	265.3 ± 0.7 (84%) ^cA^
Emmer/flaked	241.8 ± 1.9 (21%) ^B^	907.5 ± 3.9 (79%) ^B^	90.8 ± 0.7 (31%) ^B^	202.2 ± 1.6 (69%) ^B^
Barley/raw	373.5 ± 3.3 (36%) ^dA^	678.1 ± 5.0 (64%) ^dA^	234.7 ± 10.4 (55%) ^dA^	195.8 ± 1.3 (45%) ^dA^
Barley/flaked	502.6 ± 11.8 (33%) ^B^	998.8 ± 74.8 (67%) ^B^	426.8 ± 10.0 (65%) ^B^	230.5 ± 5.4 (35%) ^B^
Barley/precooked	269.0 ± 0.8 (76%) ^C^	86.1 ± 1.9 (24%) ^C^	224.8 ± 0.7 (96%) ^C^	9.3 ± 0.2 (4%) ^C^
Oat/raw	271.8 ± 12.2 (28%) ^bA^	684.6 ± 14.5 (72%) ^dA^	159.2 ± 5.9 (43%) ^aA^	215.0 ± 1.9 (57%) ^eA^
Oat/flaked	285.6 ± 2.2 (22%) ^B^	1035.9 ± 25.7 (78%) ^B^	119.4 ± 0.9 (37%) ^B^	203.0 ± 1.6 (63%) ^B^
Millet/raw	180.7 ± 1.6 (23%) ^eA^	596.6 ± 5.9 (77%) ^eA^	76.7 ± 0.2 (29%) ^eA^	192.5 ± 1.0 (71%) ^dA^
Millet/flaked	114.8 ± 0.9 (22%) ^B^	404.0 ± 3.1 (78%) ^B^	72.1 ± 0.5 (39%) ^B^	113.6 ± 0.9 (61%) ^B^
Millet/precooked	75.3 ± 0.8 (13%) ^C^	523.7 ± 14.0 (87%) ^C^	40.1 ± 0.4 (18%) ^C^	181.4 ± 4.8 (82%) ^C^
**Legumes**				
Peas/raw	181.1 ± 2.5 (69%) ^eA^	80.9 ± 6.4 (31%) ^fA^	88.4 ± 0.3 (65%) ^fA^	48.1 ± 2.1 (35%) ^fA^
Peas/flaked	163.8 ± 0.7 (72%) ^B^	64.2 ± 0.3 (28%) ^B^	99.3 ± 0.4 (89%) ^B^	12.8 ± 0.1 (11%) ^B^
Peas/precooked	174.4 ± 1.6 (56%) ^C^	136.4 ± 1.2 (44%) ^C^	103.7 ± 0.9 (77%) ^C^	31.4 ± 0.3 (23%) ^C^
Chickpeas/raw	266.3 ± 45.8 (72%) ^bA^	105.3 ± 4.7 (28%) ^gA^	121.2 ± 18.1 (63%) ^gA^	72.6 ± 7.0 (37%) ^gA^
Chickpeas/flaked	158.4 ± 3.6 (65%) ^B^	86.11.9 (35%) ^B^	59.4 ± 1.3 (86%) ^B^	9.3 ± 0.2 (14%) ^B^
Chickpeas/precooked	140.2 ± 0.9 (72%) ^C^	55.5 ± 0.4 (28%) ^C^	66.4 ± 0.4 (85%) ^C^	11.5 ± 0.1 (15%) ^C^

Within the same column, the values marked with the different letters are significantly different: lowercase letters compare the significant differences between raw flours (Tukey’s test; *p* < 0.05), and capital letters compare the significant differences between raw/flaked/precooked flours for the same species (Student’s *t*-test or Bonferroni test; *p* < 0.05).

**Table 5:** Pearson correlation coefficients (r) of polyphenol content, flavonoid content, and antioxidant capacity (TEAC) measured using the ABTS and DPPH methods in hydrophilic free and bound fractions and as total content calculated as sum of the two fractions.

	TPC	TFC	TEAC/ABTS
**Free fraction**			
TFC	0.378 *		
TEAC/ABTS	0.861 ****	0.332 ^ns^	
TEAC/DPPH	0.816 ****	0.477 *	0.802 ****
**Bound fraction**			
TFC	0.884 ****		
TEAC/ABTS	0.961 ****	0.902 ****	
TEAC/DPPH	0.951****	0.898 ****	0.956 ****
**Total (calculated)**			
TFC	0.757 ****		
TEAC/ABTS	0.956 ****	0.757 ****	
TEAC/DPPH	0.937 ****	0.762 ****	0.908 ****

**** *p* < 0.0001; * *p* < 0.05; ns = not significant, *p* > 0.05.

**Table 6:** Total carotenoid contents (TCC) and α-TEAC/ABTS and α-TEAC/DPPH value assays in the lipophilic extracts, reported as mean ± SD (*n* = 27).

Flour/Processing	Total Carotenoid Content (TCC)		α-TEAC (μmol αTE/100 g)	
	β-Carotene Equivalent (mg βCE/100 g)	Lutein Equivalent (mg LE/100 g)	ABTS	DPPH
**Cereals**				
Soft wheat mix/raw	0.330 ± 0.004 ^aA^	0.238 ± 0.003 ^aA^	117.7 ± 3.5 ^aA^	37.1 ± 2.0 ^aA^
Soft wheat mix/flaked	0.437 ± 0.006 ^B^	0.298 ± 0.004 ^B^	100.0 ± 11.0 ^A^	60.8 ± 2.9 ^B^
Einkorn/raw	1.083 ± 0.004 ^bA^	0.837 ± 0.006 ^bA^	138.1 ± 6.4 ^bA^	33.5 ± 1.1 ^bA^
Einkorn/flaked	0.251 ± 0.001 ^B^	0.159 ± 0.001 ^B^	99.9 ± 0.9 ^B^	102.6 ± 0.4 ^B^
Einkorn/precooked	2.601 ± 0.008 ^C^	1.264 ± 0.001 ^C^	105.0 ± 4.7 ^C^	27.1 ± 0.2 ^C^
Emmer/raw	0.373 ± 0.013 ^cA^	0.253 ± 0.013 ^cA^	163.4 ± 5.4 ^cA^	39.1 ± 0.8 ^cA^
Emmer/flaked	0.331 ± 0.027 ^B^	0.197 ± 0.021 ^B^	140.2 ± 2.6 ^B^	47.1 ± 0.1 ^B^
Barley/raw	0.407 ± 0.004 ^dA^	0.284 ± 0.003 ^dA^	224.8 ± 2.6 ^dA^	98.8 ± 3.6 ^dA^
Barley/flaked	0.336 ± 0.026 ^B^	0.216 ± 0.017 ^B^	218.1 ± 2.6 ^B^	135.8 ± 1.6 ^B^
Barley/precooked	0.603 ± 0.018 ^C^	0.340 ± 0.011 ^C^	216.0 ± 1.0 ^B^	103.3 ± 0.5 ^C^
Oat/raw	0.463 ± 0.016 ^eA^	0.283 ± 0.010 ^dA^	154.7 ± 0.1 ^eA^	82.6 ± 1.3 ^eA^
Oat/flaked	0.211 ± 0.006 ^B^	0.145 ± 0.004 ^B^	138.4 ± 4.3 ^B^	42.3 ± 0.5 ^B^
Millet/raw	0.759 ± 0.007 ^fA^	0.562 ± 0.005 ^eA^	55.2 ± 1.9 ^fA^	21.4 ± 1.4 ^fA^
Millet/flaked	0.104 ± 0.008 ^B^	0.057 ± 0.005 ^B^	50.3 ± 6.0 ^A^	30.1 ± 0.4 ^B^
Millet/precooked	0.334 ± 0.025 ^C^	0.221 ± 0.024 ^C^	39.4 ± 2.4 ^C^	20.5 ± 0.3 ^A^
**Legumes**				
Peas/raw	2.193 ± 0.010 ^gA^	2.286 ± 0.002 ^fA^	68.2 ± 0.5 ^gA^	27.6 ± 1.2 ^gA^
Peas/flaked	0.683 ± 0.011 ^B^	0.493 ± 0.008 ^B^	41.4 ± 3.1 ^B^	22.4 ± 0.1 ^B^
Peas/precooked	2.771 ± 0.022 ^C^	1.471 ± 0.006 ^C^	57.4 ± 4.5 ^C^	29.1 ± 0.4 ^C^
Chickpeas/raw	1.342 ± 0.008 ^hA^	0.934 ± 0.002 ^gA^	60.9 ± 0.6 ^hA^	34.3 ± 0.1 ^bA^
Chickpeas/flaked	0.282 ± 0.012 ^B^	0.167 ± 0.008 ^B^	26.2 ± 0.6 ^B^	26.1 ± 0.3 ^B^
Chickpeas/precooked	1.453 ± 0.016 ^C^	1.036 ± 0.016 ^C^	43.8 ± 0.1 ^C^	44.0 ± 0.2 ^C^

Within the same column, the values marked with the different letters are significantly different: lowercase letters compare the significant differences between raw flours (Tukey’s test; *p* < 0.05), and capital letters compare the significant differences between raw/flaked/precooked flours for the same species (Student’s *t*-test or Bonferroni test; *p* < 0.05).

**Table 7:** Chlorophyll *a*, chlorophyll *b*, and total chlorophylls (*a* + *b*) in raw, flaked, and precooked pea flours, reported as mean ± SD (*n* = 27). The chlorophyll *a* and *b* ratio is also reported.

Flour/Processing	Chlorophyll *a* (mg/100 g)	Chlorophyll *b* (mg/100 g)	Chlorophyll *a* + *b* (mg/100 g)	Chlorophyll *a*/*b*
	**Chlorophylls (actually present)**			
Peas/raw	2.92 ± 0.10 ^a^	1.79 ± 0.01 ^a^	4.72 ± 0.11 ^a^	1.6
Peas/flaked	0.21 ± 0.03 ^b^	0.041 ± 0.001 ^b^	0.25 ± 0.03 ^b^	5.1
Peas/precooked	0.24 ± 0.01 ^b^	0.21 ± 0.01 ^c^	0.45 ± 0.01 ^c^	1.1
	**Estimation of chlorophylls before pretreatments** **(chlorophylls + pheophytins)**			
Peas/raw	3.96 ± 0.07 ^c^	2.60 ± 0.02 ^d^	6.55 ± 0.08 ^d^	1.5
Peas/flaked	3.70 ± 0.07 ^d^	1.71 ± 0.01 ^e^	5.41 ± 0.08 ^e^	2.2
Peas/precooked	3.64 ± 0.01 ^d^	1.86 ± 0.01 ^f^	5.50 ± 0.02 ^e^	2.0

Within the same column, the values marked with different letters are significantly different (Tukey’s test; *p* < 0.05).
